# Elastic fiber alterations and calcifications in calcific uremic arteriolopathy

**DOI:** 10.1038/s41598-023-42492-5

**Published:** 2023-09-19

**Authors:** Hester Colboc, Philippe Moguelet, Dominique Bazin, Emmanuel Letavernier, Chenyu Sun, Anatole Chessel, Priscille Carvalho, Catherine Lok, Anne-Sophie Dillies, Guillaume Chaby, Hervé Maillard, Diane Kottler, Elisa Goujon, Christine Jurus, Marine Panaye, Ellie Tang, Philippe Courville, Antoine Boury, Jean-Benoit Monfort, François Chasset, Patricia Senet, Marie-Claire Schanne-Klein

**Affiliations:** 1 Sorbonne Université, Hôpital Rothschild, Service Plaies et Cicatrisation, UMRS_1155, 5, Rue Santerre, 75012 Paris, France; 2 Sorbonne Université, Hôpital Tenon, Anatomie et Cytologie Pathologiques, Paris, France; 3 Université Paris-Saclay, CNRS, Institut de Chimie Physique, 91405 Orsay, France; 4 Sorbonne Université, Hôpital Tenon, Service des Explorations Fonctionnelles Multidisciplinaires, UMRS_1155, Paris, France; 5 Laboratoire d’Optique et Biosciences, CNRS, Inserm, Ecole Polytechnique, Institut Polytechnique de Paris, Palaiseau, France; 6 Centre Hospitalier Universitaire de Rouen, Service de Dermatologie, Rouen, France; 7 Centre Hospitalier Universitaire d’Amiens, Service de Dermatologie, Amiens, France; 8 Private Practice, Estrées Deniécourt, France; 9 Centre Hospitalier du Mans, Service de Dermatologie, Le Mans, France; 10 Centre Hospitalier Universitaire de Caen, Service de Dermatologie, Caen, France; 11 Centre Hospitalier de Chalon-sur-Saône, Service de Dermatologie, Chalon, France; 12 Clinique du Tonkin, Service de Médecine Vasculaire, Villeurbanne, France; 13 Centre Hospitalier Universitaire de Rouen, Anatomie et Cytologie Pathologiques, Rouen, France; 14 Sorbonne Université, Faculté de Médecine, Service de Dermatologie et Allergologie, Hôpital Tenon, Paris, France; 15 Sorbonne Université, Faculté de Médecine, Service de Dermatologie 3t Allergologie, Hôpital Tenon, INSERM U1135, CIMI, Paris, France

**Keywords:** Diseases, Pathogenesis, Microscopy, Biophotonics

## Abstract

Calcific uremic arteriolopathy (CUA) is a severely morbid disease, affecting mostly dialyzed end-stage renal disease (ESRD) patients, associated with calcium deposits in the skin. Calcifications have been identified in ESRD patients without CUA, indicating that their presence is not specific to the disease. The objective of this retrospective multicenter study was to compare elastic fiber structure and skin calcifications in ESRD patients with CUA to those without CUA using innovative structural techniques. Fourteen ESRD patients with CUA were compared to 12 ESRD patients without CUA. Analyses of elastic fiber structure and skin calcifications using multiphoton microscopy followed by machine-learning analysis and field-emission scanning electron microscopy coupled with energy dispersive X-ray were performed. Elastic fibers specifically appeared fragmented in CUA. Quantitative analyses of multiphoton images showed that they were significantly straighter in ESRD patients with CUA than without CUA. Interstitial and vascular calcifications were observed in both groups of ESRD patients, but vascular calcifications specifically appeared massive and circumferential in CUA. Unlike interstitial calcifications, massive circumferential vascular calcifications and elastic fibers straightening appeared specific to CUA. The origins of such specific elastic fiber’s alteration are still to be explored and may involve relationships with ischemic vascular or inflammatory processes.

## Introduction

Uremic calciphylaxis, also called calcific uremic arteriolopathy (CUA), is a rare and severely morbid condition that predominantly affects dialyzed end-stage renal disease (ESRD) patients^1^. Characteristic histologic features of CUA include vascular calcifications, subcutaneous adipose tissue and dermis micro-vessel thrombosis, often accompanied by extravascular calcifications^2^. Various risk factors of CUA have been identified, including female sex, diabetes mellitus, higher body mass index, vitamin K antagonist treatments and elevated serum calcium, phosphorus, and parathyroid hormone levels^3^.

Increased serum calcium-phosphate product is a common complication of ESRD and leads to tissular calcifications^4^. Multiple studies have reported both vascular and interstitial skin calcifications in ESRD patients without CUA, showing that their presence is not specific to the disease and that other phenomena are involved in its pathogenesis^5–7^.

We previously reported that CUA calcifications are composed of pure calcium–phosphate apatite, always circumferential in small to medium-sized vessels, with interstitial deposits in most cases^8^. Despite well-characterized clinical, histological and ultra-structural descriptions of CUA, its precise pathogenic mechanism remains unclear. Skin calcifications are also observed in many other skin diseases, including inflammatory, genetic or infectious diseases^9^. Remarkably, they are not always associated with abnormal calcium and phosphorus levels but often occur following an inflammatory process, suggesting a link between inflammation and calcification^10^. This association has been largely demonstrated in cardio-vascular diseases, leading to calcification of nucleation sites within vascular elastic fibers^11, 12^. Some authors therefore suspected that, in CUA pathogenesis, inflammation may cause specific alterations of dermal and vascular elastic fibers through the action of matrix metalloproteinase, leading to the creation of mineralization nucleation sites^13, 14^. Exploring these structural alterations could help understand CUA pathogenesis and find appropriate therapeutic approaches.

The aim of this study was to compare elastic fiber structure and skin calcifications in ESRD patients with CUA (ESRD + CUA) to those without CUA (ESRD) using innovative methods.

## Materials and methods

### Case selection and histopathological analyses

This retrospective case–control study included 14 adults diagnosed with CUA from a previous study, confirmed according to Hayashi’s criteria, seen in six French hospitals between January 2006 and January 2017^8, 15^. Local diagnoses done at each hospital were blindly confirmed by a central dermatopathologist who reviewed all samples. For comparison, clinical, biological and histological data of 12 ESRD patients’ without CUA were also analyzed. These controls underwent skin resection for benign or malignant skin tumors (negative margins were then analyzed) or skin biopsy for non-CUA inflammatory lesions. Except for two controls, for which skin samples were collected from the trunk, both ESRD + CUA and ESRD samples were collected from the lower limb.

For each sample, 4-µm–thick sections of paraffin-embedded skin biopsies were stained with haematoxylin–eosin–saffron (HES), Orcein, and Yasue, allowing visualization of elastin and calcifications.

### Multiphoton microscopy

Multiphoton microscopy provides simultaneous mapping of a variety of tissue components by combining various modes of contrast^16–24^. Intrinsic fluorescent components, including elastic fibers, can be visualized in the fluorescence channel, while fibrillar collagen is specifically visualized using Second Harmonic Generation (SHG) without any staining.

Seven-µm–thick sections of paraffin-embedded skin biopsies were deposited on glass slides and directly imaged without any staining using a custom-built upright multiphoton microscope, with 860 nm circularly-polarized laser excitation and high numerical aperture (1.05) 25 × water-immersion objective lens (XLPLN-MP, Olympus)^18^. SHG and fluorescence were detected in 2 different channels using appropriate spectral filters as previously described^18^. Series of images were recorded from the epidermis to the hypodermis using a motorized stage and stitched together to get a complete mapping of the skin structure. Endogenous fluorescence was obtained from various skin components including elastin fibers and calcifications^16–24^.

### Quantitative analysis of elastic fibers

Quantitative comparison of elastic fiber structure in ESRD + CUA cases versus ESRD was performed on multiphoton images using the open-source machine-learning software Ilastik^25^. Elastic fibers were segmented in 2–5 images of 510 × 510 µm^2^ acquired in the mid-dermis for each sample, resulting in 975 ± 510 segmented fibers per sample. The smallest elastin fibers were discarded from further analysis because they correspond mainly to fibers whose orientation is almost perpendicular to the section plane or that are highly curved and therefore appear small in the 2D section despite their true size. This resulted in 513 ± 302 fibers per sample when keeping only fibers with total area larger than 100 pixels, i.e., 17.6 µm^2^. Such automated segmentation of elastin fibers was successfully obtained only for subgroups of 7/14 ESRD + CUA and 8/12 ESRD samples. The other samples could not be segmented automatically because they showed a low contrast, presumably because of different fixation durations or methods. This low contrast resulted in an increased background noise in the multiphoton images and impeded any automatic segmentation.

Two structural parameters were calculated for each segmented fiber using the “Regionprops” function in MATLAB: the major axis length and the eccentricity of the ellipse fitting the elastic fiber. The eccentricity is defined as the ratio of the distance between the center and each focus of the ellipse to the half-length of the major axis; it is 0 for a circle and tends towards 1 for a flat ellipse. The major axis length measures the length of the fibers in the 2D section, which may be smaller than the real length in the 3D tissue. This limitation does not apply to the eccentricity as it is a ratiometric measure.

### Field-emission scanning electron microscopy coupled with energy dispersive X-ray

These techniques provide the ultrastructural characteristics of skin tissue, including elastic fibers and calcifications, as well as the subcellular location of the latter at the micrometer scale^25^. Supplementary 4-µm-thick sections of paraffin-embedded skin biopsies were deposited on low-e microscope slides (MirrIR, Kevley Technologies, U.S.A.) for field-emission scanning electron microscopy (FE-SEM) and energy dispersive X-ray (EDX) (Zeiss SUPRA55-VP, Oberkochen, Germany). As previously described, high-resolution images were obtained by two secondary electron detectors: an in-lens SE detector and an Everhart Thornley SE detector^26^. In some samples, EDX was also used to confirm the calcic nature of the sub-micrometric deposits. Measurements were taken at low voltage (1–2 kV) without the usual carbon coating of the sample surface.

### Statistical analyses

Statistical analysis was performed on clinical, biological and structural parameters to compare ESRD + CUA to ESRD patients using Prism (Graphpad). Data were expressed as median [range] or number (percentage). Chi-2 test with Yates’ correction was used to compare qualitative variables. Mann–Whitney test was used to compare quantitative variables. Multiple t-tests based on the Holm-Sidak method were used for elastin fiber structural parameters, resulting in an adjusted p-value.

### Ethical approval

This study was performed in compliance with Good Clinical Practices and the Declaration of Helsinki, and in accordance with French law. Formal Ethics Committee approval of the study protocol was obtained (no. NCT05112744). The patients in this manuscript have given written informed consent to participate in the study.

### Informed consent

The patients in this manuscript have given written informed consent to participate in the study. The study was conducted ethically in accordance with the World Medical Association Declaration of Helsinki.

## Results

### Clinical and biological findings

Fourteen ESRD + CUA were compared to twelve ESRD patients. As summarized in the Table 1, ESRD + CUA and ESRD patients had comparable demographic, comorbidities and biological data except for corrected serum calcium levels, which were statistically higher in ESRD + CUA patients (2.59 mmol/L^−1^ versus 2.36 mmol/L^−1^, *p* = 0.031)^27^. CUA was mostly distal (11 patients, 79%), presenting as skin necrosis (10 patients, 71%) or ulceration (8 patients, 57%), livedo reticularis (7 patients, 50%), indurated plaques (6 patients, 43%) or nodular lesions (3 patients, 21%). ESRD patients underwent skin resection for skin tumors in 7 cases and skin biopsy for non CUA inflammatory lesions in 5 cases: 2 pigmented purpuric dermatosis, 2 inflammatory nodular lesions (histological examination revealed calcinosis cutis) and one violaceous nodular lesions (histological examination revealed adiponecrosis).

**Table 1:** Baseline clinical, biological and histological characteristics of ESDR + CUA and ESDR patients.

Characteristic	ESDR + CUA (n = 14)	ESDR (n = 12)	*p* value
Demographic			
Female/male ratio	2.5	1.4	0.77
Age at diagnosis, years	67 [56–79]	65 [45–70]	0.22
Co-morbidity			
Dialysis	10 (71%)	12 (100%)	0.15
Dialysis-to-skin biopsy interval (months)	15 [4–23]	21 [6–45]	0.32
Diabetes	9 (64%)	4 (33%)	0.24
Hypertension	14 (100%)	8 (67%)	0.071
Body mass index > 30 kg/m^−2^	5 (36%)	4 (33%)	0.77
Vitamin K antagonists	4 (29%)	3 (25%)	0.81
Biological			
Serum calcium, mmol/L^−1^	2.25 [2.12–2.48]	2.17 [2.02–2.36]	0.39
Corrected serum calcium, mmol/L	2.59 [2.41–2.91]	2.36 [2.29–2.55]	**0.031**
Serum phosphate, mmol/L^−1^	2.13 [1.15–2.73]	2.06 [1.43–2.70]	0.59
Calcium x phosphate, mmol^−2^/L^−2^	4.98 [2.68–6.11]	4.51 [3.12–5.99]	0.98
Calcium × phosphate > 4.5 mmol^−2^/L^−2^	8 (57%)	6 (50%)	0.98
Serum albumin, g/L^−1^	28.5 [23.4–31.8]	31.8 [29.1–33.1]	0.083
Interstitial calcification			
Adipocytes’ membrane	13 (93%)	2 (17%)	**0.0007**
Dermic elastic fibers	11 (79%)	3 (25%)	**0.019**
Sweat glands’ basal lamina	5 (36%)	7 (58%)	0.55
Vascular calcifications			
Voluminous and circumferential	14 (100%)	0	** < 0.0001**
Small-sized vessels	14 (100%)	6 (50%)	**0.011**
Medium-sized vessels	6 (43%)	4 (33%)	0.93
Thrombosis	3 (21%)	0	0.28

Values are expressed as *n* (%) or median [interquartile range]. CUA, calcific uremic arteriolopathy.

### Analysis of elastic fibers’ structure

**Figure 1:**
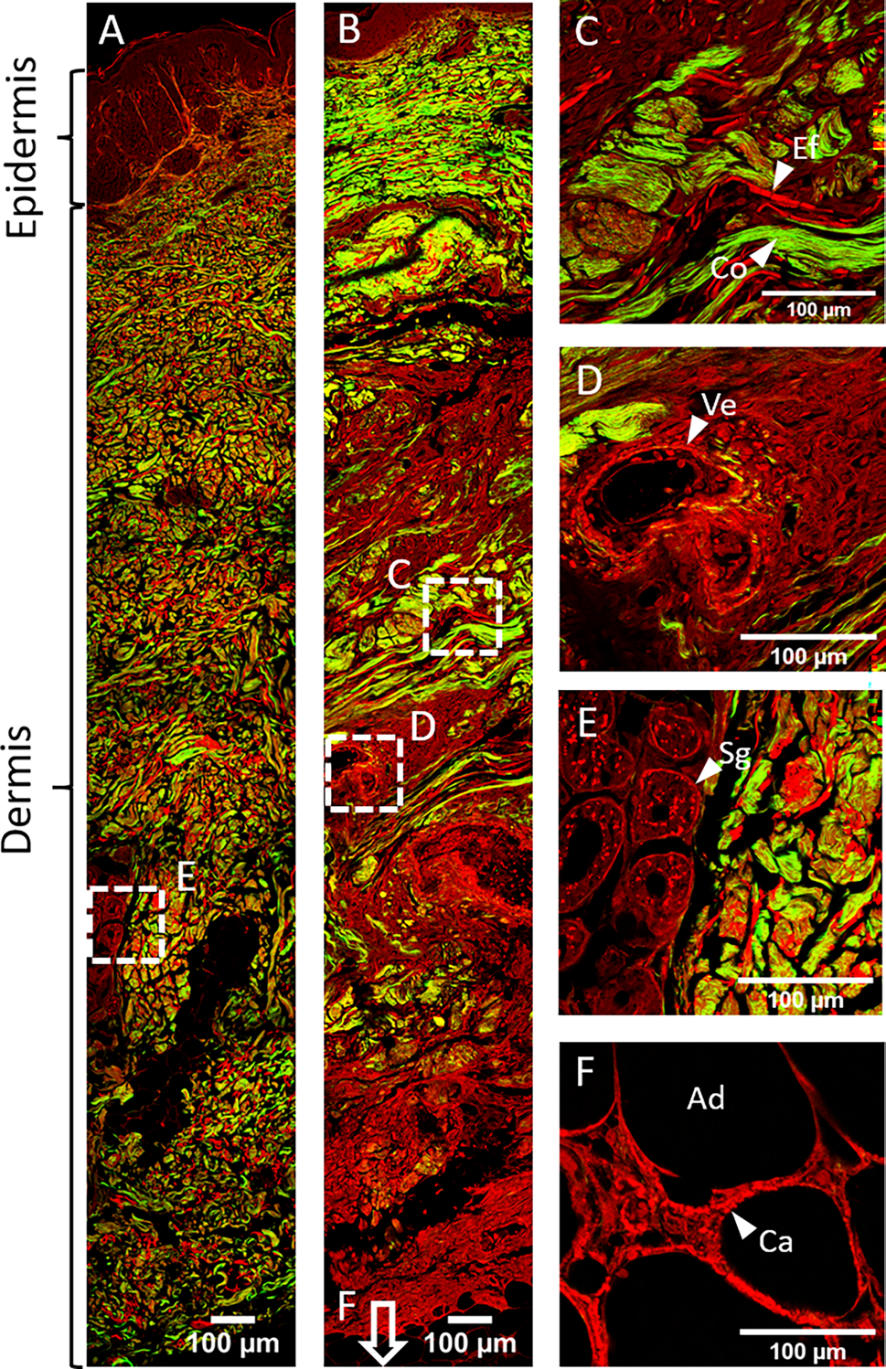
Multiphoton microscopy of unstained skin sections. (**A**) Full slice imaging of a skin biopsy of ESRD patient. (**B**) Full slice imaging of a ESRD + CUA skin biopsy showing straight and fragmented elastic fibers. (**C**) Collagen fibers (Co, in green), and straight and fragmented elastic fibers (Ef, in red) in ESRD + CUA dermis. (**D**) Medium-sized vessel (Ve) with circumferential vascular calcifications in ESRD + CUA dermis. (**E**) Sweat glands (Sg) in dermis of ESRD patient. (**F**) Calcifications (Ca) of an adipocyte (Ad)’s membrane in ESRD + CUA hypodermis. All the images are obtained by merging fluorescence (in red) and SHG (in green) images recorded simultaneously in two detection channels. Elastic fibers, calcifications, sweat glands and adipocytes membrane are visualized through their intrinsic fluorescence, fibrillar collagen is visualized by SHG.

Multiphoton microscopy allowed a global analysis of the samples (Fig. 1A,B) by recording simultaneously the endogenous fluorescence of elastic fibers and skin calcifications and the SHG of fibrillar collagen in the same unstained sections. Calcifications were identified within the vessel walls (Fig. 1D) and in some cases along adipocyte membrane which then appeared fragmented (Fig. 1F). Sweat glands were also visualized by their intrinsic fluorescence (Fig. 1E). CUA elastic fibers often appeared straight and somewhat fragmented, (Fig. 1C). Orcein staining consistently showed the same pattern of elastin fibers (Supplementary Fig. S1). This pathological aspect was clearly observed only in a subset of the ESRD + CUA samples (7 of 14 samples) but in none of the ESRD. No association could be made between clinical and biological parameters—such as severity of clinical symptoms, delayed biopsy or higher Calcium × Phosphate product—and this aspect of straight and fragmented elastic fibers. Nevertheless, an association might exist, that couldn’t be identified due to the relatively small quantity of cases in these 2 subgroups.

**Figure 2:**
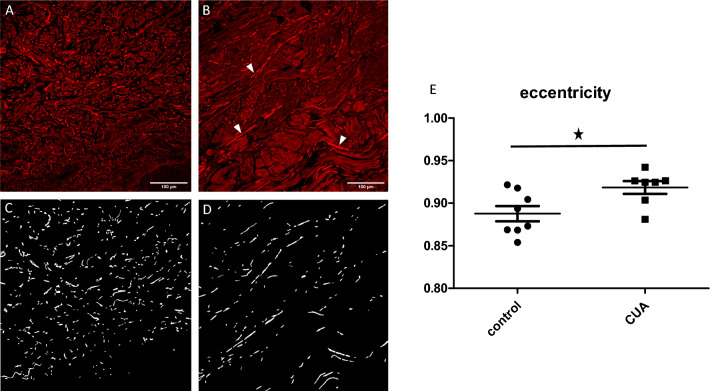
Quantitative analysis of elastic fiber structure based on their intrinsic fluorescence visualized using multiphoton microscopy. (**A**) Mid-dermis of a ESRD biopsy, showing normal elastic fibers. (**B**) Mid-dermis of a ESRD + CUA biopsy, showing straight and fragmented elastic fibers (arrow heads). (**C**) Segmentation of the elastic fibers of panel (**A**). (**D**) Segmentation of the elastic fibers of panel (**B**). (**E**) Comparison of the eccentricity of the elastic fibers between ESRD + CUA and ESRD (*p* = 0.044).

Automatic segmentation of elastic fibers was performed using Ilastik software in a subgroup of samples (7/14 ESRD + CUA and 8/12 ESRD) with well-contrasted multiphoton images (Fig. 2A,B,C,D)^25^. Quantitative analysis in Matlab then showed that the eccentricity of the elastic fibers was statistically higher in the ESRD + CUA (0.92 on average for all 7 ESRD + CUA, including samples exhibiting not clearly straight fibers) than in the 8 ESRD (0.88 on average, adjusted p-value: *p* = 0.044), meaning that elastic fibers were significantly straighter in ESRD + CUA than in ESRD (Fig. 2E). Comparison of the major dimension of the fibers (major axis length of the ellipse encompassing the fiber) showed no significant difference.

### Analysis of skin calcifications

**Figure 3:**
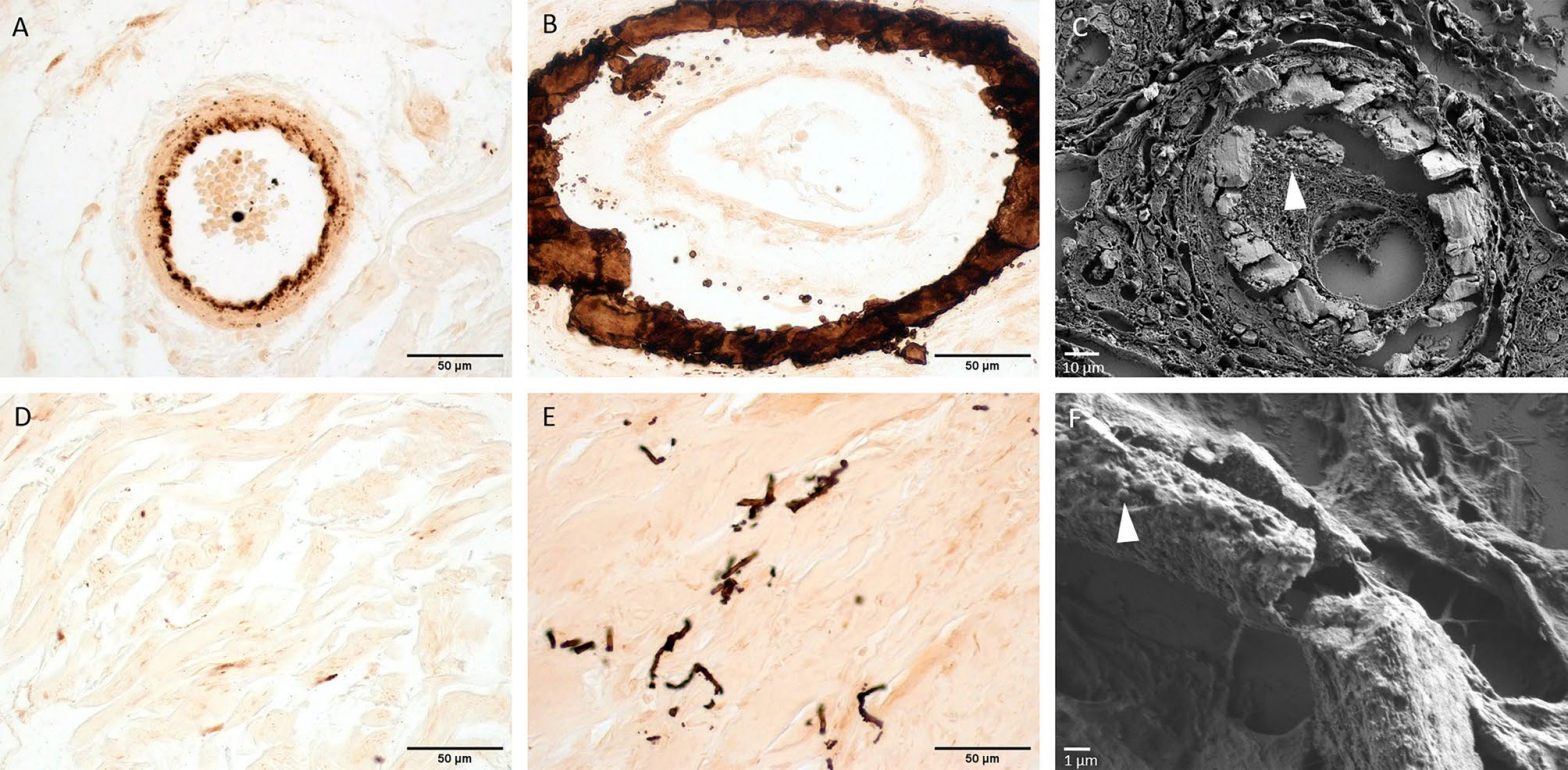
Optical microscopy of Yasue-stained sections (**A**,**B**,**D**,**E**) and FE-SEM (**C**,**F**) characterization of vessels and mid-dermis. (**A**) ESRD sample showing calcifications restricted to the internal elastic lamina of a small-sized vessel in the dermis. (**B**) ESRD + CUA sample showing voluminous and circumferential calcifications of a medium-sized vessel in the dermis. (**C**) FE-SEM of a small-sized vessel extensively calcified from a ESRD + CUA sample. Arrowhead: micrometric calcified spherules near calcified plaques. (**D**) ESRD sample and (**E**) ESRD + CUA sample showing calcification of mid-dermis elastic fibers. (**F**) FE-SEM of a fragmented elastic fiber from a ESRD + CUA sample. Arrowhead: nanometric calcified spherules, covering the surface of the elastic fibers.

Regarding vascular calcifications, consistently with what we have previously described, all ESRD + CUA samples showed massive circumferential vascular calcifications, associated with thrombosis in 3 (21%) patients (Table 1)^8^. Vascular calcifications were also observed in 6 (50%) ESRD without CUA, although they appeared slightly different, with location in the internal elastic lamina and media (i.e. Monckeberg medial calcinosis) and were not associated with thrombosis (Fig. 3A,B). Furthermore, there was significantly more small-sized vessels calcifications in ESRD + CUA patients than in ESRD (14 versus 6, *p* = 0.011).

**Figure 4:**
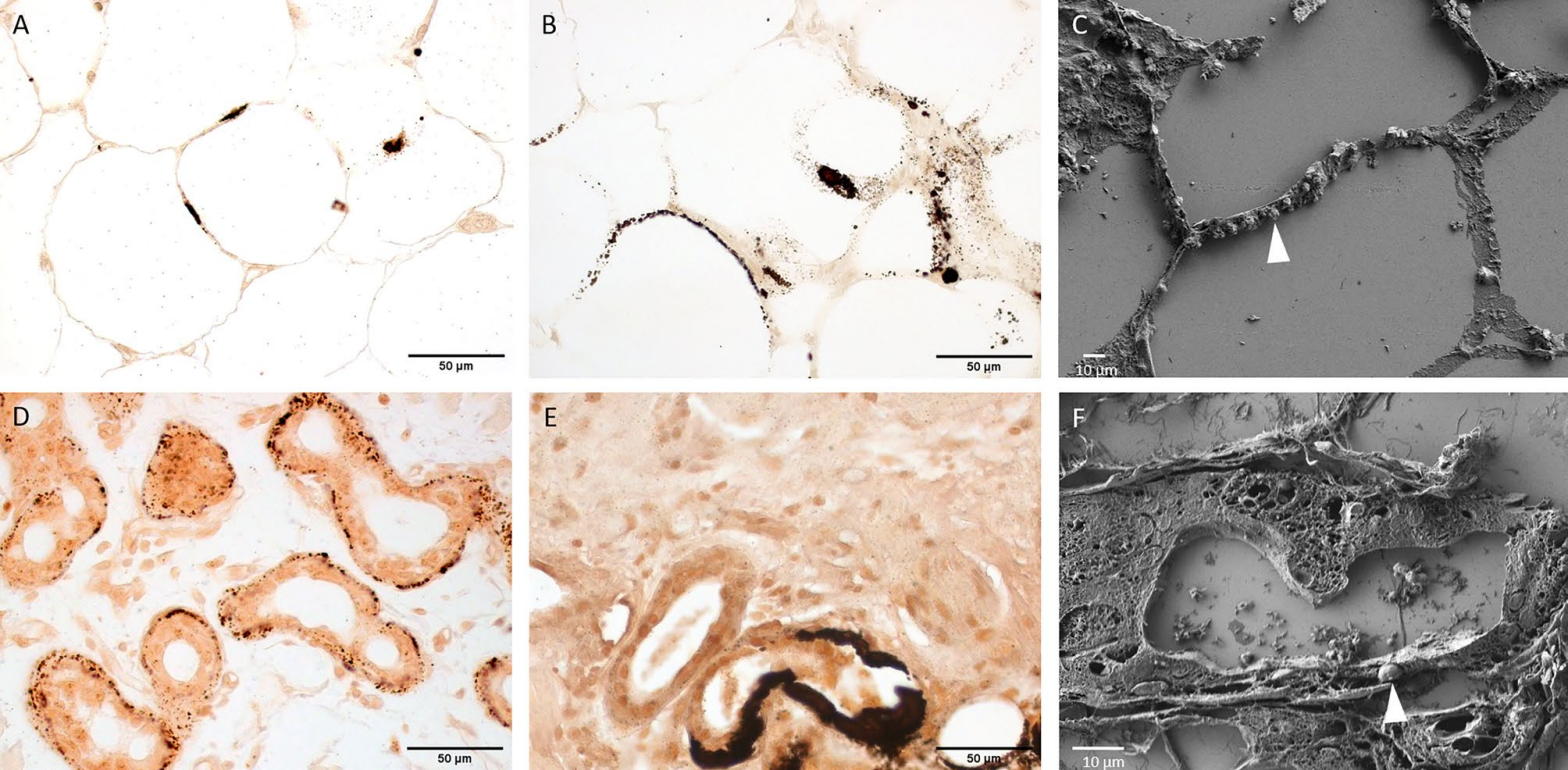
Optical microscopy of Yasue-stained sections (**A**,**B**,**D**,**E**) and FE-SEM (**C**,**F**) characterization of adipocytes and sweat glands. (**A**) ESRD sample and (**B**) ESRD + CUA sample showing calcification of adipocyte membranes. (**C**) FE-SEM of micrometric calcified spherules along the adipocyte membranes (arrowhead) in ESRD + CUA sample. (**D**) ESRD sample and (**E**) ESRD + CUA sample showing calcifications of sweat glands basal lamina. (**F**) FE-SEM of sweat glands basal lamina with micrometric calcified spherule (arrowhead) in ESRD + CUA sample.

The presence and location of interstitial calcifications were not specific to CUA: they were identified in dermal elastic fibers (Fig. 3D,E), on adipocyte membranes (Fig. 4A,B), and in the basal lamina of the sweat glands (Fig. 4D,E). However, there was significantly more calcifications of the dermal elastic fibers (11 versus 3, *p* = 0.019) and of the adipocyte membranes (13 versus 2, *p* = 0.0007) in ESRD + CUA than in ESRD (Table 1). Furthermore, as observed in vascular calcifications, interstitial calcifications were globally larger in ESRD + CUA than in ESRD.

### Ultrastructural analysis

The ultrastructure of the vascular and interstitial calcifications was similar in ESRD + CUA and ESRD: they consisted of small calcic spherules of 500 nm to 1 µm, which were identified on adipocyte membranes and within the basal lamina of the sweat glands (Figs. 3C, 4C,F and supplementary Fig. S2). However, the micrometric structural organization of these spherules was completely different in ESRD + CUA and ESRD vessels: they were regrouped into large plaques in ESRD + CUA but only coalescent without plaque formation in ESRD vessels (Fig. 3C and supplementary Fig. S2).

FE-SEM coupled with EDX also showed that fragmented straight elastic fibers were covered with smaller spherical entities of 300 to 500 nm, composed of Phosphorus and Calcium (Fig. 3F and supplementary Fig. S2).

## Discussion/conclusion

In this study, we identified alterations of elastic fibers and aspects of skin calcifications that are specific to CUA. We demonstrated that elastic fibers were straighter in ESRD + CUA than in ESRD, which has not been reported before. Furthermore, we confirmed that large vascular circumferential calcifications were highly specific to CUA patients and preferentially located in small-sized vessels^8^.

These results suggest that CUA may occur as a consequence of the association of phosphocalcic disorders and specific alterations of elastic fibers, making the latter prone to calcify. A specific trigger may occur in ESRD + CUA patients, leading to the straightening of the elastic fibers and to large calcifications, in contrast with limited calcifications in ESRD patients, despite comparable risk factors for CUA. This potential trigger is still to be explored, specifically its link with inflammation^12, 13^. Further studies including more patients would also be interesting in order to explore the link between the clinical findings and the structural aspect of the elastic fibers.

A previous study has shown that in vitro, fragmentation of the elastic fibers promotes their calcification^28^. Interestingly, in our study, although elastic fibers appeared fragmented on 7 ESRD + CUA, we did not identify fragmentation from a quantitative approach, as the axis length of the elastic fibers was comparable in ESRD + CUA and ESRD. This may result from the low sensitivity in the measurement of a 3D parameter from a 2D section (see Methods).

The chronological link between the straightening of the elastic fibers and the occurrence of calcifications is still to be determined. Indeed, we cannot exclude that straightening of the elastic fibers may be the consequence of former calcifications of the surface of the elastic fibers. However, among the 7 ESRD + CUA with straight elastic fibers, only 3 had dermal calcifications. Furthermore, 3 ESRD also had dermal calcifications, but none of them had straight elastic fibers. Taken together, these results suggest that straight elastic fibers may not be a consequence of their calcifications but possibly the initial phenomenon, later promoting calcification.

The review performed by Bahrani et al. also highlights the fact that very few studies focus on interstitial tissue modification and rather analyze skin calcifications^29^. Some groups identified—in small case series or in case reports—curled, frayed, calcified and basophilic elastic fibers located in the septa of the subcutaneous fat and described these as pseudoxanthoma elasticum-like changes^30–32^. However, only 5 cases of CUA were analyzed in this case series, without any control group, and the study was only qualitative. This might explain why our results are different, as we found straighter elastic fibers in ESRD + CUA than in ESRD. Furthermore, we cannot exclude that different types of elastic fiber alterations could lead to their calcifications.

Our study showed that calcifications in CUA occurred mainly within skin structures rich in elastin: vessel walls, dermis and basal lamina of sweat glands^33^. Bahrani et al. showed, using classic optical microscopy, that calcifications of subcutaneous small vessels, thrombosis and perieccrine calcifications are more specific to CUA^29^. Our results are consistent with those of Bahrani’s review, except that we did not observe a significant difference in sweat glands basal membrane calcifications in ESRD + CUA compared to ESRD.

Using FE-SEM coupled to EDX, we were also able to characterize the ultra-structure of the elastic fibers and calcifications and confirmed the existence of nanometric calcified spherules in both ESRD + CUA and ESRD, which were not visible using optical microscopy because of their size. Such nanometric calcifications are likely to represent the very early stages of the calcification process and are therefore very interesting to characterize in order to provide, hopefully in a near future, new therapeutic tools. Identification of such small deposits also questions the limits associated with the different techniques used here. We indeed cannot exclude that even FE-SEM failed to identify nanometric-calcified spherules and that they were present in more ESRD partients than we described. This hypothesis strengthens our results regarding the elastic fibers structural alterations, highlighting once again the pathological specificity of this feature and the non-specificity of some of the skin calcifications. However, although location and ultra-structural aspect of the calcifications were similar in ESRD + CUA and in ESRD, their micrometric organization was different. In ESRD + CUA, multiples spherules regrouped into large calcified plaques—as previously described in other dystrophic skin calcifications –, while coalescence of calcified spherules was observed in ESRD patients but did not lead to the formation of such large calcified plaques^34^. This difference might be explained by a greater quantity of both interstitial and vascular calcifications in ESRD + CUA than in ESRD.

As many clinical and biological parameters were comparable in both groups, including serum phosphate and calcium phosphate products, the presence of large calcifications in CUA cases cannot exclusively be explained by systemic mechanisms and local parameters should be taken into consideration, such as elevated local concentration of organic supports—phospholipid or DNA –, favoring calcic nucleation^35–37^. Quite interestingly, CUA cases were more likely to present small-sized vessels calcification and adipocyte membrane calcifications. We can hypothesize that calcifications of small sized vessels of the hypodermis may lead to more frequent adiponecrosis in ESRD + CUA than in ESRD, in turn resulting in local elevation of phospholipid concentration and increased peri-adipocyte membrane calcifications. This hypothesis is consistent with previous studies, suggesting that CUA-specific calcifications are primarily located into subcutaneous fat^38, 39^. Further analysis using Raman and nano-infrared spectroscopy might provide a better understanding of the molecular environment in which these large calcifications are formed^40^.

Our innovative results were obtained using complementary techniques: histology, multiphoton microscopy, FE-SEM, and EDX. Multiphoton microscopy has been used previously in other skin diseases in which elastic fibers are altered including pseudoxanthoma elasticum and solar elastosis but not in CUA^17, 20, 21, 23, 41, 42^. This technique advantageously provides simultaneous visualization of all major components of the skin, including collagen and elastic fibers, calcifications, sweat glands and adipocyte membranes. Our automated segmentation approach provides a robust and unbiased (blinded) means to analyze the alterations of the elastic fibers in ESRD + CUA compared to ESRD and may be generalized to other diseases in which elastic fibers are altered. Such an automated segmentation is implemented in a straightforward way in grey-level images with sufficient contrast obtained by multiphoton microscopy, but it may be generalized to colored histological images from orcein-stained sections and enable quantification of elastic fibers using classical optical microscopy. It may also be generalized to 3D multiphoton images in thick unstained samples and enable robust and accurate measurements of the length of elastic fibers.

In conclusion, our histological, multiphoton microscopy, FE-SEM, and EDX analyses provide a better understanding of the pathogenesis of CUA. We identified specific alterations of elastic fiber structure as well as voluminous and circumferential vascular calcifications in ESRD + CUA, not observed in ESRD. Although diffused calcifications were observed in both groups, they appeared much larger in ESRD + CUA than in ESRD. Furthermore, dermal elastic fibers, adipocyte membranes, and small-sized vessels were significantly more calcified in ESRD + CUA than in ESRD. Further studies, analyzing the potential presence of organic supports promoting calcifications should be performed to provide a better understanding of this multifactorial pathology.

### Supplementary Information

Supplementary Information 1.

Supplementary Information 2.

Supplementary Information 3.

## Data Availability

Data supporting the findings of this study are available from the corresponding author on reasonable request.

## References

[1] Weenig RH, Sewell LD, Davis MD, McCarthy JT, Pittelkow MR (2007). Calciphylaxis: Natural history, risk factor analysis, and outcome. J. Am. Acad. Dermatol..

[2] Nigwekar SU, Thadhani R, Brandenburg VM (2018). Calciphylaxis. N. Engl. J. Med..

[3] Nigwekar SU (2016). A nationally representative study of calcific uremic arteriolopathy risk factors. J. Am. Soc. Nephrol..

[4] Block GA (2000). Prevalence and clinical consequences of elevated Ca x P product in hemodialysis patients. Clin. Nephrol..

[5] Cassius C (2018). Calciphylaxis in haemodialysed patients: Diagnostic value of calcifications in cutaneous biopsy. Br. J. Dermatol..

[6] Ellis CL, O’Neill WC (2018). Questionable specificity of histologic findings in calcific uremic arteriolopathy. Kidney Int..

[7] Nigwekar SU, Nazarian RM (2018). Cutaneous calcification in patients with kidney disease is not always calciphylaxis. Kidney Int..

[8] Colboc H (2019). Localization, morphologic features, and chemical composition of calciphylaxis-related skin deposits in patients with calcific uremic arteriolopathy. JAMA Dermatol..

[9] Reiter N, El-Shabrawi L, Leinweber B, Berghold A, Aberer E (2011). Calcinosis cutis: Part I Diagnostic pathway. J. Am. Acad. Dermatol..

[10] Lotz M (1995). Interleukin 1 beta suppresses transforming growth factor-induced inorganic pyrophosphate (PPi) production and expression of the PPi-generating enzyme PC-1 in human chondrocytes. Proc. Natl. Acad. Sci. USA.

[11] Gourgas O, Muiznieks LD, Bello DG, Nanci A, Sharpe S, Cerruti M (2019). Cross-linked elastin-like polypeptide membranes as a model for medial arterial calcification. Biomacromol.

[12] Vyavahare N, Jones PL, Tallapragada S, Levy RJ (2000). Inhibition of matrix metalloproteinase activity attenuates tenascin-C production and calcification of implanted purified elastin in rats. Am. J. Pathol..

[13] Munavalli G, Reisenauer A, Moses M, Kilroy S, Arbiser JL (2003). Weight loss-induced calciphylaxis: Potential role of matrix metalloproteinases. J. Dermatol..

[14] Basalyga DM, Simionescu DT, Xiong W, Baxter BT, Starcher BC, Vyavahare NR (2004). Elastin degradation and calcification in an abdominal aorta injury model: Role of matrix metalloproteinases. Circulation.

[15] Hayashi M (2013). Calciphylaxis: Diagnosis and clinical features. Clin. Exp. Nephrol..

[16] Koenig K, Riemann I (2003). High-resolution multiphoton tomography of human skin with subcellular spatial resolution and picosecond time resolution. J. Biomed. Opt..

[17] Koehler MJ, Preller A, Elsner P, König K, Hipler UC, Kaatz M (2012). Non-invasive evaluation of dermal elastosis by in vivo multiphoton tomography with autofluorescence lifetime measurements. Exp. Dermatol..

[18] Bancelin S (2015). Ex vivo multiscale quantitation of skin biomechanics in wild-type and genetically-modified mice using multiphoton microscopy. Sci. Rep..

[19] Lynch B (2017). How aging impacts skin biomechanics: A multiscale study in mice. Sci. Rep..

[20] Le Digabel J, Houriez-Gombaud-Saintonge S, Filiol J, Lauze C, Josse G (2018). Dermal fiber structures and photoaging. J. Biomed. Opt..

[21] Jones JD, Ramser HE, Woessner AE, Quinn KP (2018). In vivo multiphoton microscopy detects longitudinal metabolic changes associated with delayed skin wound healing. Commun. Biol..

[22] Fast A (2020). Fast, large area multiphoton exoscope (FLAME) for macroscopic imaging with microscopic resolution of human skin. Sci. Rep..

[23] Pena AM (2022). In vivo multiphoton multiparametric 3D quantification of human skin aging on forearm and face. Sci. Rep..

[24] Baugh LM (2017). Non-destructive two-photon excited fluorescence imaging identifies early nodules in calcific aortic-valve disease. Nat. Biomed. Eng..

[25] Berg S (2019). Ilastik: interactive machine learning for (bio)image analysis. Nat. Methods.

[26] Brisset, F. Microscopie Électronique à Balayage et Microanalyses. EDP Sciences (2012).

[27] Parent X, Spielmann C, Hanser AM (2009). "Corrected" calcium: Calcium status underestimation in non-hypoalbuminemic patients and in hypercalcemic patients. Ann. Biol. Clin..

[28] Boraldi F, Moscarelli P, Lofaro FD, Sabia C, Quaglino D (2020). The mineralization process of insoluble elastin fibrillar structures: Ionic environment vs degradation. Int. J. Biol. Macromol..

[29] Bahrani E, Perkins IU, North JP (2020). Diagnosing calciphylaxis: A review with emphasis on histopathology. Am. J. Dermatopathol..

[30] Penn LA, Brinster N (2018). Calciphylaxis with pseudoxanthoma elasticum-like changes: A case series. J. Cutan. Pathol..

[31] Lewis KG, Lester BW, Pan TD, Robinson-Bostom L (2006). Nephrogenic fibrosing dermopathy and calciphylaxis with pseudoxanthoma elasticum-like changes. J. Cutan. Pathol..

[32] Chen EL, Altman I, Braniecki M (2020). A Helpful Clue to Calciphylaxis: Subcutaneous Pseudoxanthoma Elasticum–like Changes. Am. J. Dermatopathol..

[33] Spicer SS, Brissie RM, Thompson NT (1975). Variability of dermal elastin visualized ultrastructurally with iron hematoxylin. Am. J. Pathol..

[34] Colboc H (2022). Calcified leg ulcers in older patients: Clinical description, morphology, and chemical characterization. J. Gerontol. A Biol. Sci. Med. Sci..

[35] Satsangi N, Satsangi A, Glover R, Ong JL, Satsangi RK (2004). Osteoblast response and calcium deposition on phospholipid modified surfaces. J. Mater. Sci. Mater. Med..

[36] Coscas R (2017). Free DNA precipitates calcium phosphate apatite crystals in the arterial wall in vivo. Atherosclerosis.

[37] Bazin D, Letavernier E, Haymann JP, Frochot V, Daudon M (2020). Les pathologies cristallines humaines: les premières étapes de la pathogénèse. Ann. Biol. Clin..

[38] Ng AT, Peng DH (2011). Calciphylaxis. Dermatol. Ther..

[39] Williams EA, Moy AP, Cipriani NA, Nigwekar SU, Nazarian RM (2019). Factors associated with false-negative pathologic diagnosis of calciphylaxis. J. Cutan. Pathol..

[40] Deinsberger J, Felhofer M, Kläger JP, Petzelbauer P, Gierlinger N, Weber B (2021). Raman spectroscopy reveals collagen and phospholipids as major components of hyalinosis in the arteriolosclerotic ulcer of Martorell. J. Eur. Acad. Dermatol. Venereol..

[41] Tong PL (2013). A quantitative approach to histopathological dissection of elastin- related disorders using multiphoton microscopy. Br. J. Dermatol..

[42] Wang H, Shyr T, Fevola MJ, Cula GO, Stamatas GN (2018). Age-related morphological changes of the dermal matrix in human skin documented in vivo by multiphoton microscopy. J. Biomed. Opt..

