# Supplementary figures and images for: Elastic fiber alterations and calcifications in calcific uremic arteriolopathy
Source: Sci Rep. 2023 Sep 19;13:15519. doi: 10.1038/s41598-023-42492-5 (PMC10509184; doi:10.1038/s41598-023-42492-5)

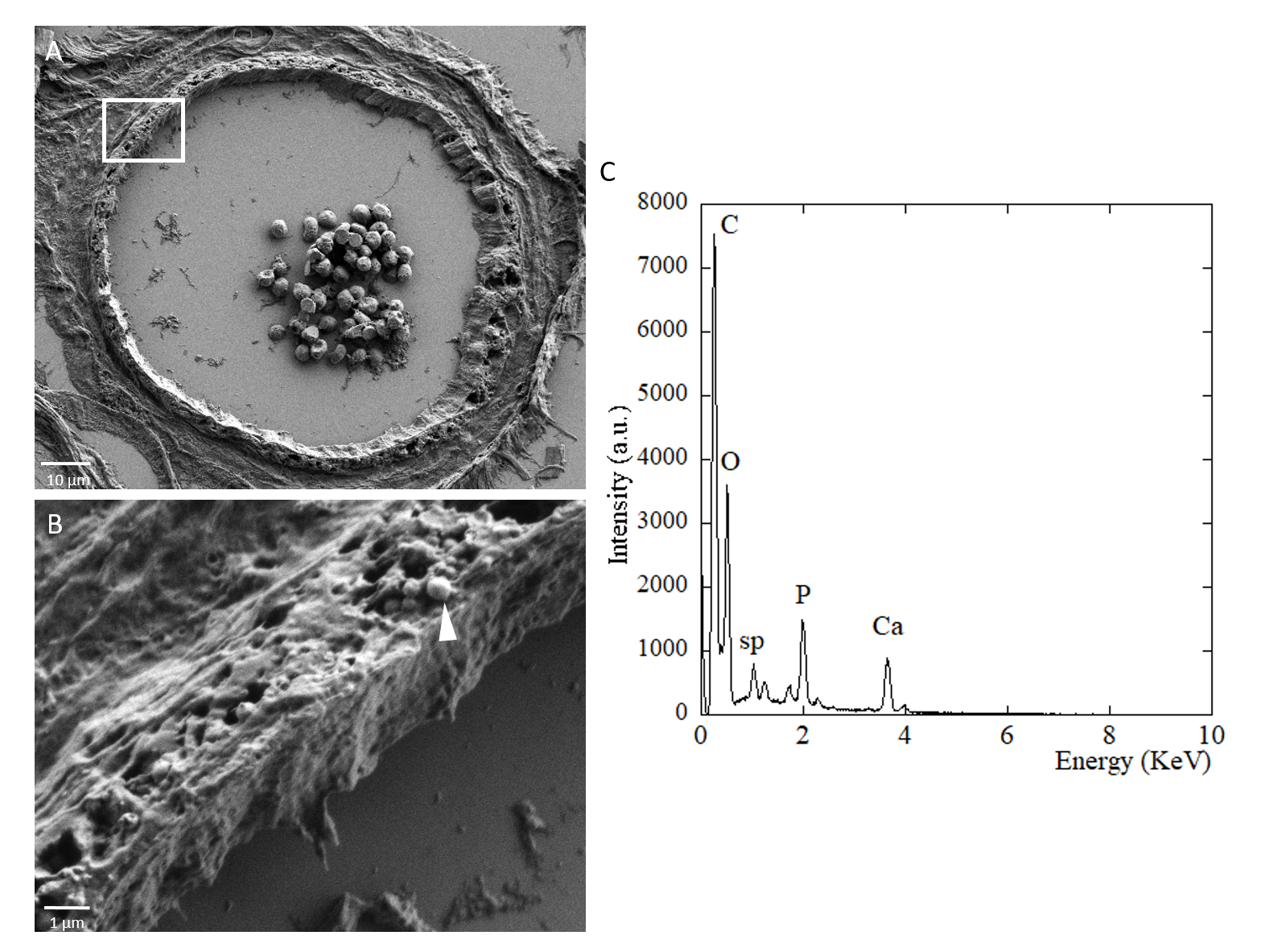

Supplement: Supplementary file 1 — Supplementary Information 1. [file 41598_2023_42492_MOESM1_ESM.tif]

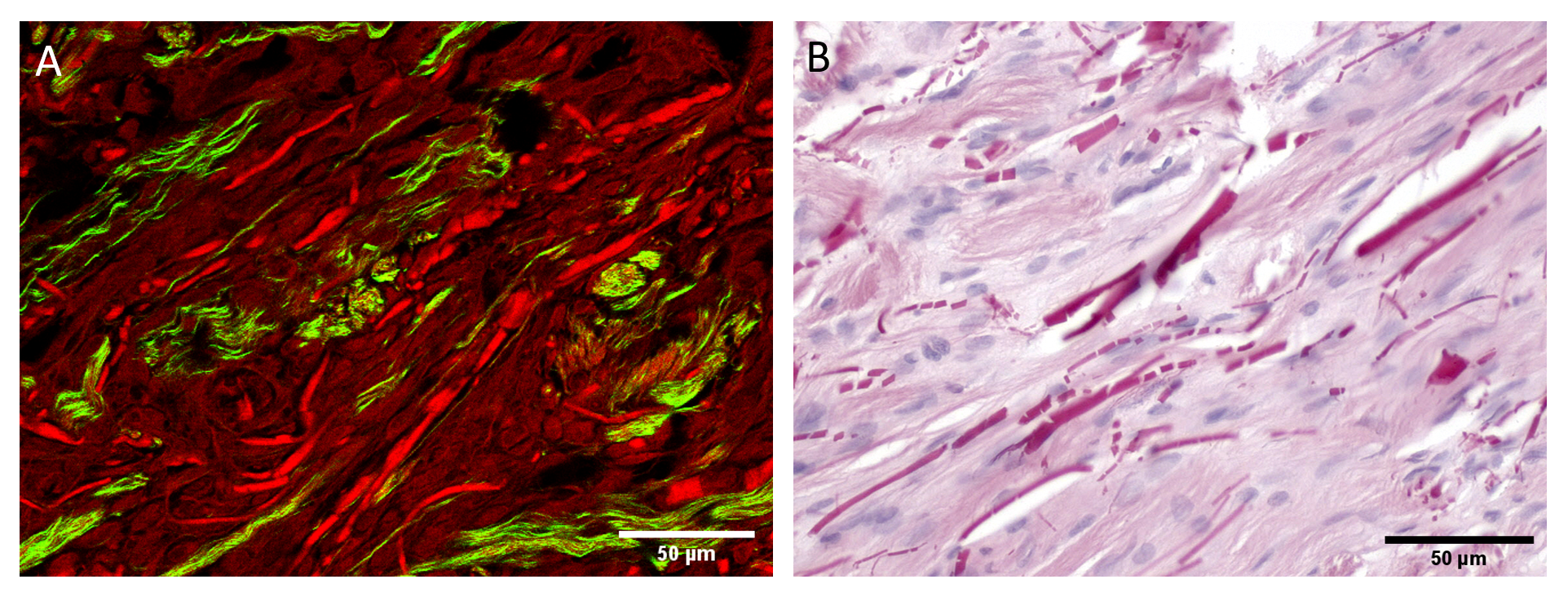

Supplement: Supplementary file 2 — Supplementary Information 2. [file 41598_2023_42492_MOESM2_ESM.tif]
