# Supplementary material for: Elastic fiber alterations and calcifications in calcific uremic arteriolopathy
Source: Sci Rep. 2023 Sep 19;13:15519. doi: 10.1038/s41598-023-42492-5 (PMC10509184; doi:10.1038/s41598-023-42492-5)
Supplement: Supplementary file 3 — Supplementary Information 3. [file 41598_2023_42492_MOESM3_ESM.docx]

Supplementary Figure S1: comparison of multiphoton and histological imaging of elastic fibers. A. Multiphoton microscopy of an unstained ESRD + CUA sample with straight and fragmented elastic fibers. B. Classic optical imaging of an Orcein-stained section of the same sample.

Supplementary Figure S2: FE-SEM and EDX characterization of a ESRD skin sample. A. Calcified medium-sized vessel, with diffused spherules within the vessel wall. B. Higher magnification of the white rectangle on panel A, showing multiple spherules of 300 to 500 nm (arrowhead). C. EDX analyses confirming the Calcium (Ca, K Æ 3.691 keV, K Æ 4.012 keV) and Phosphate (P, K Æ 2.014 keV) composition of these nanometric spherules. Contributions of Carbon (C, K Æ 0.277 keV), Oxygen (O, K Æ 0.525 keV) and of a sum peak (SP) due to the coincidence of two O K photons can also be identified.
